# Short‐lived neutralizing activity against SARS‐CoV‐2 in newborns of immunized mothers

**DOI:** 10.1111/pai.70084

**Published:** 2025-04-09

**Authors:** Marta Stracuzzi, Claudia Vanetti, Micaela Garziano, Maida Micheloni, Maria Luisa Murno, Gian Vincenzo Zuccotti, Mario Clerici, Vania Giacomet, Daria Trabattoni

**Affiliations:** ^1^ ASST Fatebenefratelli Sacco, Paediatric Infectious Disease Unit Milan Italy; ^2^ Department of Pathophysiology and Transplantation University of Milan Milan Italy; ^3^ Department of Biomedical and Clinical Sciences Univeristy of Milan Milan Italy; ^4^ Department of Paediatrics Ospedale Dei Bambini Vittore Buzzi Milan Italy; ^5^ IRCCS Fondazione Don Gnocchi Milan Italy

**Keywords:** hybrid immunity, neutralizing activity, newborns, passive immunity, SARS‐CoV‐2

## Abstract

**Background:**

Newborns under 6 months of age are at high risk of hospitalization for acute respiratory failure following SARS‐CoV‐2 infection. Herein, we analyzed neonatal protection against SARS‐CoV‐2 passively acquired after mother vaccination and/or infection (hybrid immunity).

**Methods:**

We enrolled seventy‐eight newborns of immunized mothers against SARS‐CoV‐2 before or during pregnancy, through vaccination and/or infection. Infants were stratified based on the anamnestic lack (SARS‐CoV‐2 Vaccinated – SV)/presence (SARS‐CoV‐2 Infected and Vaccinated – SIV) of COVID‐19 maternal infection. SARS‐CoV‐2‐specific Neutralizing Activity (NA) in plasma was assessed by virus neutralization assay (vNTA) against the SARS‐CoV‐2 Omicron strain at delivery (T0), 3 and 6 months after birth (T3 and T6). Cytokine and chemokine profiles in newborns were also analyzed.

**Results:**

At birth, significantly lower NA was observed in infants of SV compared to that of SIV mothers; NA declined equally in both groups 3 months after delivery. The presence of at least 4 immunizing events in the mother significantly enhances the NA against SARS‐CoV‐2 in newborns, regardless of the type of immunization (vaccination or hybrid immunity) and of the timing of the last maternal immunization. Finally, cytokines and chemokines plasma levels were high at birth in all newborns, followed by a decline over the subsequent month.

**Conclusion:**

Our findings suggest that, independently of a previous SARS‐CoV‐2 infection or vaccination, it is reasonable to upgrade the recommendation of a booster dose during pregnancy to a “strongly recommended” status, with a view to conferring protection to newborns in the first months after delivery.

**Abbreviations**
AGA: Adequate for Gestational Age
CPE: cytopathic effect
LGA: Large for Gestational Age
NA: neutralizing activity
SGA: Small for Gestational Age
SIV: SARS‐CoV‐2 Infected and Vaccinated
SV: SARS‐CoV‐2 Vaccinated
TCID50: 50% tissue culture infectious dose
vNTA: virus neutralization assay
WHO: World Health Organization

**Key message**

Despite the recommendation for SARS‐CoV‐2 vaccination during pregnancy, vaccine hesitancy persists among pregnant women, even though newborns under the age of 6 months are at high risk of hospitalization for acute respiratory failure following SARS‐CoV‐2 infection. The results of our study demonstrated clear advantages of maternal vaccination in infant protection. The public health and medical communities should ensure that pregnant women receive appropriate immunization upgrading the current recommendation for SARS‐CoV‐2 vaccination from “recommended” to “strongly recommended”.

## 1 INTRODUCTION

Following SARS‐CoV‐2 infection, infants are more likely to require hospitalization for acute respiratory failure than older children, especially with the upsurge of new variants.^1^ It is noteworthy that, during the period in which Omicron was considered the primary variant of concern, the risk of neonatal death was elevated among newborns of unvaccinated mothers.^2^ Since newborns under the age of 6 months are not yet eligible for vaccination, prevention of infection and severe illness remains an issue of significant concern within this age group. In this context, maternal vaccination may be a useful tool, as maternal immunoglobulin G (IgG) antibodies can cross the placental barrier, thereby providing both pregnant women and their offspring with protection. Thus, immunization during pregnancy is an efficient strategy and is currently limited to tetanus, pertussis, and influenza vaccines.^3^ Recently, the Centers for Disease Control and Prevention (CDC) has also recommended the administration of the respiratory syncytial virus (RSV) vaccine for pregnant women, and a 2023 study demonstrated that vaccination against RSV during pregnancy can effectively reduce the incidence of infant RSV illness.^4^

Concerning COVID‐19 vaccination during pregnancy or in women who are breastfeeding, the CDC has recently recommended a booster dose of the COVID‐19 vaccine. In this regard, SARS‐CoV‐2‐specific antibodies were found in cord blood, breast milk, and serum specimens obtained from infants,^5^, ^6^, ^7^ indicating the transfer of maternal antibodies to them. Furthermore, there is evidence that a booster COVID‐19 vaccine dose during pregnancy is not associated with increased risks of neonatal adverse events^8^ and the risk of preterm birth and adverse neonatal outcomes is reduced among neonates of vaccinated mothers.^2^ Regarding maternal anti‐SARS‐CoV‐2 antibodies, when comparing antibodies produced after vaccination or infection, infants born to vaccinated mothers have higher levels of anti‐spike antibodies than those whose mothers developed antibodies from a natural infection during pregnancy.^9^ Nevertheless, the role of vaccination following SARS‐CoV‐2 infection (hybrid immunity) in pregnant women remains to be elucidated. It has been shown that hybrid immunity results in the production of higher levels of neutralizing antibodies in both maternal and umbilical cord blood at delivery, compared with unimmunized pregnant women.^10^ These results are consistent with data from the general population, where hybrid immunity reinforced protection against reinfection and severe COVID‐19.^11^, ^12^ This consideration notwithstanding, the clinical significance of maternal hybrid immunity in COVID‐19 protection and its duration in newborns is unclear. Herein, we aimed to estimate the effectiveness of maternal SARS‐CoV‐2 vaccination on mounting neutralizing activity in a cohort of newborns within 6 months of life, born from women that had received a different number of vaccine doses and had or had not experienced SARS‐CoV‐2 infection.

## 2 MATERIALS AND METHODS

### 2.1 Patient population

This is a prospective observational study on newborns and infants born between May and July 2023 at the Luigi Sacco Hospital, Milan, Italy, from women who were either infected with and/or vaccinated against SARS‐CoV‐2. The study received approval from the ethical committee of the coordinating center in Milan (protocol number 0034645). All research was performed in accordance with relevant regulations, and informed consent was obtained from all the participants' parents or legal guardians. The study was performed in accordance with the Declaration of Helsinki.

The mothers' history of SARS‐CoV‐2 infection was documented based on any past positive result of molecular or antigen tests from nasal/pharyngeal swab specimens, either during or before pregnancy. Data regarding clinical symptoms or signs and details of COVID‐19 vaccination, including the number and type of shots received, were also collected. None of the mothers tested positive for SARS‐CoV‐2 at the time of delivery.

We enrolled 78 newborns (38 females and 40 males) with a mean gestational age of 38.74 weeks (range 35^+5^–41^+5^ weeks). Newborns were stratified based on the mothers' history of COVID‐19: 31 newborns from SARS‐CoV‐2 Vaccinated (SV) mothers and 47 from SARS‐CoV‐2 Infected and eventually Vaccinated (SIV) mothers.

For each enrolled newborn, clinical data and 2 mL of peripheral blood were collected at three time points: at birth (T0), at 3 months of age (T3), and at 6 months of age (T6). Plasma for neutralization assays and cytokine analysis was obtained by centrifugation of whole blood at 400 ***g*** for 10 min and stored at −20°C until use.

Anthropometric data of all newborns, including birth weight, length, and head circumference, were recorded and compared with the World Health Organization (WHO) Child Growth Standards at birth, 3 months, and 6 months of age.

Other parameters indicative of neonatal health were also evaluated, such as APGAR score and umbilical cord arterial pH. The APGAR scores were determined at 1, 5, and 10 min for all 78 newborns.

Newborns were stratified based on the mothers' history of COVID‐19: 31 newborns from SARS‐CoV‐2 Vaccinated (SV) mothers and 47 from SARS‐CoV‐2 Infected and Vaccinated (SIV) mothers.

### 2.2 Plasma neutralization assay (NA)

Plasma samples were thawed at room temperature and then heat‐inactivated for 30 min at 56°C. Neutralization activity (NA) against the SARS‐CoV‐2 variant Omicron (BA.1) was assessed using a virus neutralization assay (vNTA)^13^ at four different time points: T0, T1, T2, and T3. In brief, plasma samples were serially diluted two‐fold starting at 1:10 and mixed with an equal volume of the tissue culture infectious dose 50 (TCID50) of the SARS‐CoV‐2 variant Omicron. All dilutions were prepared in DMEM High Glucose (Euroclone, Milan, Italy) supplemented with 2 mM L‐Glutamine, 100 U/mL of penicillin and streptomycin (Life Technologies, Carlsbad, USA), and 2% fetal bovine serum (FBS) (Euroclone, Milan, Italy). The plasma‐virus mixtures were incubated for 2 h at 37°C in a humidified environment with 5% CO2. Post incubation, each dilution was transferred in duplicate to a 96‐well microplate pre‐seeded with 2 × 10^4^ VeroE6 cells (CRL‐1586™, African green monkey kidney epithelial cells, ATCC®, Manassas, VA, USA). The plates were then incubated for 72 h at 37°C with 5% CO_2_. After incubation, VeroE6 cells were examined to determine the degree of cytopathic effects (CPE) compared to the virus control. The neutralizing titer was defined as the highest dilution that resulted in a 90% reduction of CPE. A positive titer was defined as equal to or greater than a 1:10 dilution, and dilutions were reported as absolute values in the graphs. Each test included controls for plasma (1:10 dilution), cells (VeroE6 cells alone), and the virus (TCID50, three‐fold serial dilution).

### 2.3 Multiplex cytokine analyses

A multiplex assay for 17 cytokines was conducted on plasma from patients using a Luminex magnetic bead immunoassay (Bio‐Rad, CA, USA),^14^ following the manufacturer's instructions.

### 2.4 Statistical analyses

Two‐way ANOVA was applied for non‐parametric multiple comparisons. A *p*‐value <.05 was chosen as the cutoff for significance. Data were analyzed with GraphPad Prism 9.

## 3 RESULTS

### 3.1 Neonatal and maternal characterization

The average maternal age at the time of delivery was 33.96 years (95% CI 32.55–35.37), with 47.46% (*n* = 28) of vaginal deliveries and 52.54% (*n* = 31) of caesarean sections.

The mean birth weight was 3192.44 g (95% CI: 3082.87–3302.00) and it was considered adequate for 85.9% of the newborns. Based on gestational age, 78.21% of newborns were classified as Adequate for Gestational Age (AGA) (*n* = 61), 16.67% as Small for Gestational Age (SGA) (*n* = 13) and 5.13% as Large for Gestational Age (LGA) (*n* = 4). The mean birth length was 48.99 cm (95% CI 48.55–49.44), and the mean head circumference at birth was 34.3 cm (95% CI 33.98–34.63).

The mean score at 1 min post‐delivery was 9.182 (95% CI: 9.02–9.34), with 97.44% of neonates (*n* = 76) with a normal APGAR score (>7), and 9.909 (95% CI: 9.84–9.97) at 5 min. At 10 min, 97.44% (*n* = 76) of neonates had an APGAR score of 10. Three cases showed slow adaptation to extrauterine life, but no newborn exhibited signs of asphyxia or required neonatal resuscitation after birth. The umbilical cord arterial pH, assessed using point‐of‐care analysis for all the newborns enrolled, had a mean value of 7.31 (95% CI: 7.28–7.33), ranging from 7.12 to 7.44. None of the newborns in the analyzed cohort had a pH below 7.10, the pH threshold for adverse neurological outcomes.

**FIGURE 1:**
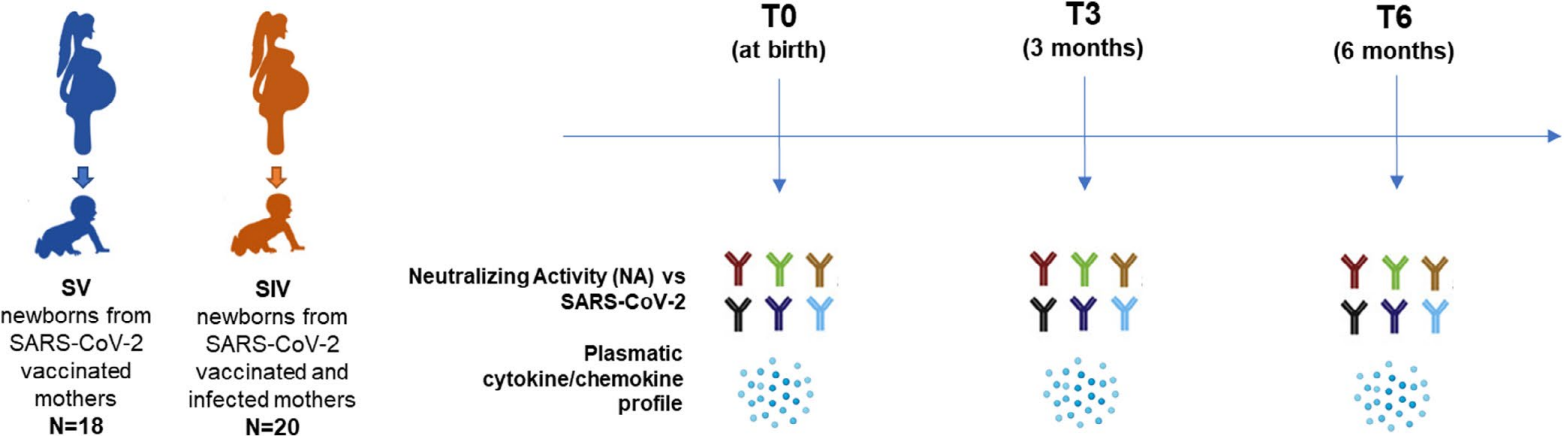
Timeline of sample collection. T0: One day before vaccination; T1: 25 days after the second dose; T2: 6 months after second dose—administration of the third dose; T3: 3 months after the third dose.

Hypoglycemia was observed in one newborn (1.28%) and monitored throughout their stay in the neonatal ward. Additionally, two newborns (2.56%) developed jaundice and underwent phototherapy with complete resolution. No SARS‐CoV‐2 infections or other significant diseases were reported in the newborn cohort, and no hospitalizations linked to severe symptoms from COVID‐19 were registered. A schematic representation of the timeline of samples collection and analyses is depicted in Figure 1. Figure S1 illustrates the complexity of the description of each mother's infections and vaccinations over time.

There was a significant drop‐out rate at follow‐up, resulting in 43 patients at 3 months and 19 patients at 6 months, with a total of 19 and 7 samples analyzed at T3 and T6, respectively.

All assessed infants exhibited harmonious growth in accordance with the WHO growth charts for weight, length, and head circumference, adjusted for age and sex. Detailed data are shown in Table 1.

**TABLE 1:** Summary of Perinatal Data, Anthropometric Measurements and Feeding Practices in Study Population over time (0–3‐6 months).

	T0		
	Mean value (95% CI)		% (Number of Newborns)
Gestational Age	38.74 (38.50–38.98)	Term	94.87% (74)
		Late Preterm	5.13% (4)
Birth Weight [g]	3192.44 (3082.87–3302.00)	SGA	16.67% (13)
Crown‐Heel Length [cm]	48.99 (48.55–49.44)	AGA	78.21% (61)
Head Circumference [cm]	34.30 (33.98–34.63)	LGA	5.13% (4)
Umbelical cord pH	7.31 (7.28–7.33)	Positive Coombs test	2.63% (2)
APGAR 1′	9.18 (9.02–9.34)	Hypoglycemia	1.28% (1)
APGAR 5′	9.91 (9.84–9.97)	Jaundice	2.56% (2)

	T3	T6
	Mean Value (95% CI)	Mean Value (95% CI)
Body Weight [g]	6198.49 (5949.25–6447.73)	7587.11 (7136.78–8037.43)
Crown‐Heel Length [cm]	61.80 (60.93–62.68)	67.02 (65.21–68.82)
Head Circumference [cm]	40.59 (40.23–40.96)	43.48 (42.62–43.48)
	**% (Number of Infants)**	**% (Number of Infants)**
HU	53.49% (23)	57.89% (11)
FM	32.56% (14)	36.84% (7)
HU + FM	13.95% (6)	5.26% (1)
Weaning	/	89.47% (17)

Abbreviations: AGA, Adequate for Gestational Age; FM, Formula milk; HU, Human Milk; LGA, Large for Gestational Age; SGA, Small for Gestational Age.

Additionally, feeding practices were evaluated at 3 months for 43 infants, showing that 53.49% (*n* = 23) were exclusively breastfed, 32.56% (*n* = 14) were only bottle‐fed, and 13.95% (*n* = 6) received mixed feeding. At 6 months, 89.47% (*n* = 17) of parents reported having already begun weaning, introducing some meals per day as recommended by their primary care pediatrician, while maintaining the same type of breastfeeding for other meals.

The average time from mother's last vaccination to delivery was similar in both SV and SIV groups (mean ± SD: SV = 16 months±3.9; SIV = 16 months±3.3). In the SIV group, the average time elapsed between the last infection and delivery was 13 months (mean ± SD: 13 months±7.8). The 22.66% (*n* = 17) of the newborns were born to mothers who contracted the infection during pregnancy, 10.66% (*n* = 8) to mothers vaccinated during pregnancy, and 66.66% (*n* = 50) to mothers who had all immunizing events before pregnancy.

All the mothers who had COVID‐19 reported having either asymptomatically or with mild symptoms, with no one requiring hospitalization or oxygen therapy. The most frequently reported symptoms were fever, myalgia and joint pain, fatigue, pharyngodynia, cough, and headache.

The number of doses received ranged from 2 to 4. Of these, 10.81% of vaccinations were administered during pregnancy.

### 3.2 Neutralizing activity (NA) in newborns enrolled

Among the 78 newborns included in the study, only 38 blood samples were considered suitable to assess the plasma neutralizing activity (NA) at birth.

At the 3‐month follow‐up visit, 21 infants, accounting for 47.73% of the total, consented to undergo a new blood sample collection. Among them, a total of 19 specimens were successfully analyzed.

**FIGURE 2:**
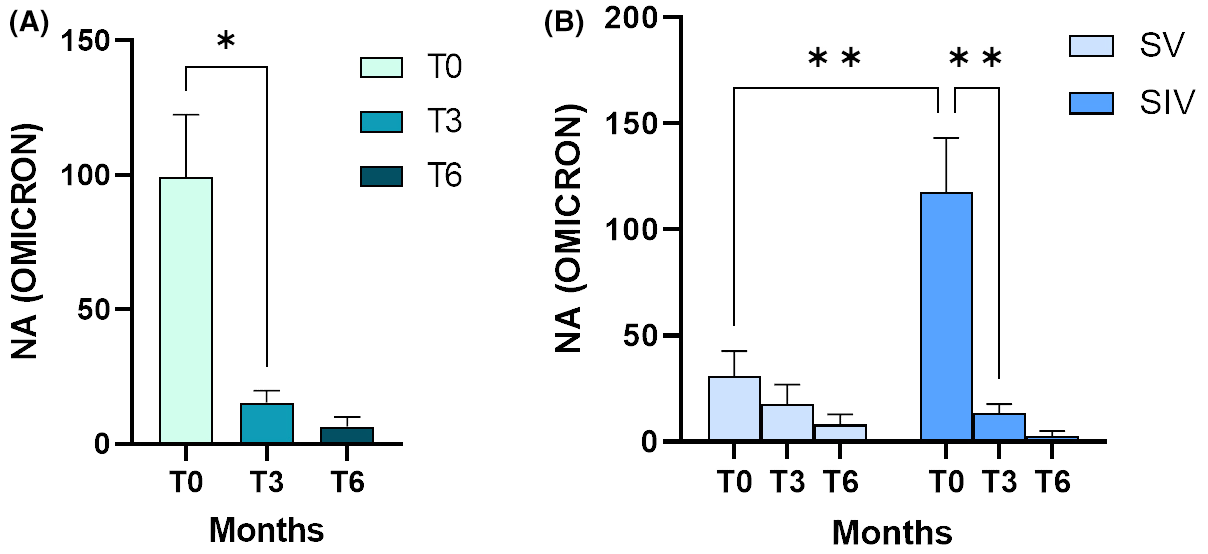
Neutralizing activity (NA) at birth newborns from SV or SIV mothers. (A) Plasma neutralizing activity (NA) over time (T0, T3 and T6) against the SARS‐CoV‐2 Omicron variant is shown in all the newborns enrolled. (B) The data of the plasma NA over time (T0, T3 and T6) against the SARS‐CoV‐2 Omicron variant is shown by dividing the newborns born from SV (light blue) and SIV (dark blue) mothers. Mean values ± SEM are reported. Significance was set at *p* < .05 (Two‐way ANOVA test, *p* values adjusted for multiple comparisons). *p < .05, **p < .01.

As depicted in Figure 2A, considering all the newborns enrolled, we observed a significant decrease in NA of 84.64% between T0 and T3 (*p* < .05), and 57.86% from T3 to T6. The NA data were analyzed by dividing the enrolled subjects into two groups, distinguished based on maternal infection, SV and SIV. At birth, infants of SV (*n* = 18) mothers displayed significantly lower NA compared to newborns of SIV (*n* = 20) mothers (*p* < .01). However, NA declined over time after delivery in both groups (T3 vs. T0 in SV = −39% ± 0.6; T3 vs. T0 in SIV = −33% ± 0.5), with a significant decline reached in the SIV group between T0 and T3 (*p* < .01). This latter event was not observed in the SV group. However, the comparison between these two cohorts at T3 and T6 showed no statistical significance, indicating that in both cohorts of infants the neutralizing activity was low at both 3 and 6 months, with no significant differences between SIV and SV. The results for the comparison of NA in the two cohorts at different follow‐up times are shown in Figure 2B.

### 3.3 NA levels according to the number of maternal immunizing events

**FIGURE 3:**
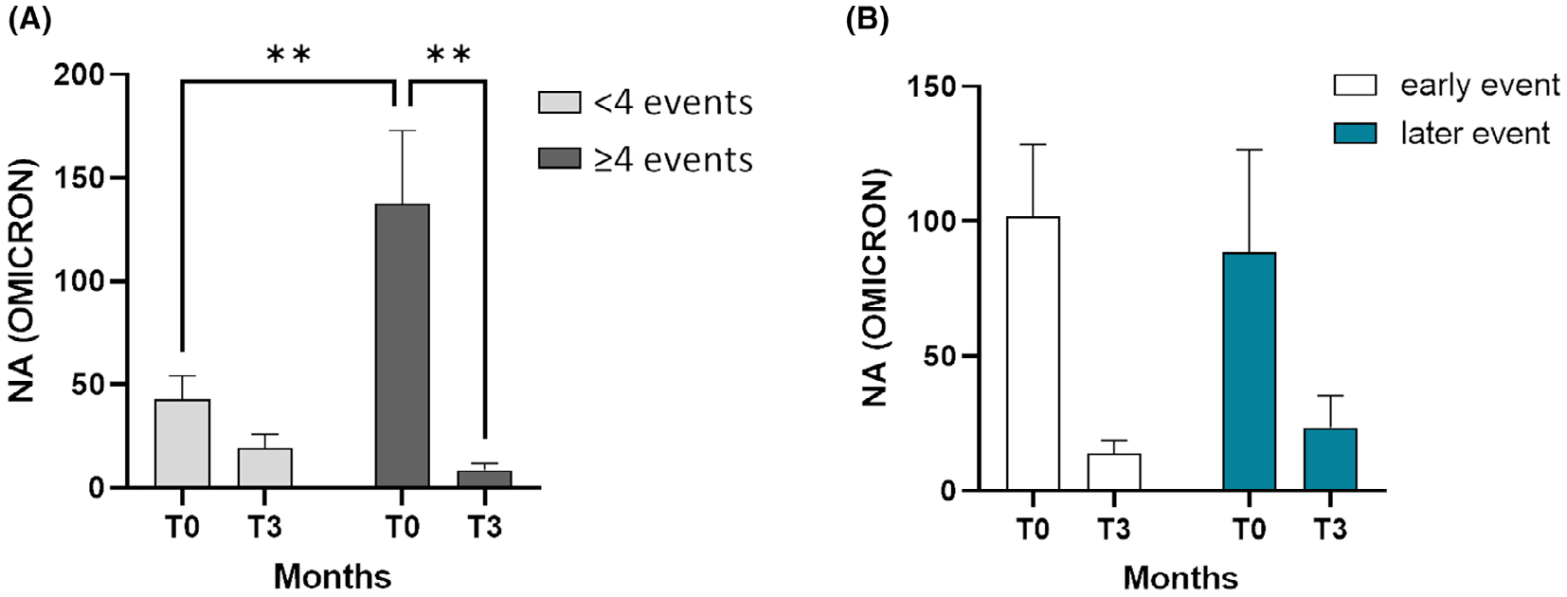
NA at birth in newborns from mothers who experienced 4 or more immunizing events. (A) Plasma NA over time (T0 and T3) against the SARS‐CoV‐2 Omicron variant is shown in newborns from mothers who experienced 3 or less immunizing events () or 4 or more immunizing events (). (B) The data of the plasma NA over time (T0 and T3) against the SARS‐CoV‐2 Omicron variant is shown by dividing the newborns born from mothers who experienced immunizing events by 12 months before delivery (early event, white) or from mothers who experienced the immunizing events within 12 months before delivery (late event, dark green). Mean values ± SEM are reported. Significance was set at *p* < .05 (Two‐way ANOVA test, *p* values adjusted for multiple comparisons). **p < .01.

Considering the benefits associated with the administration of booster doses and their impact on maternal immunity, we classified our cohort according to the total number of immunizing events experienced by each mother. The term' immunizing event' ncompasses both the administration of a single vaccine dose and the occurrence of maternal infection. We study the difference in neutralizing activity between infants whose mothers had received three or fewer immunizing events (<4 events, T0 *n* = 26, T3 *n* = 12) with that of infants whose mothers had received four or more immunizing events (≥4 events, T0 *n* = 14, T3 *n* = 7). Comparison of these two cohorts showed that the presence of 4 or more immunizing events in the mother, rather than 3 or fewer, highly significantly influences the NA against SARS‐CoV‐2 in the newborns (*p* < .01), regardless of the type of immunizing events acquired, through either vaccination or infection. However, values at T3 were very low in both groups. The results are depicted in Figure 3A. To understand whether the timing of immunization events could also somehow influence the NA levels, the population of infants was again divided into two groups: infants with the last maternal immunization event occurring before 12 months prior to birth were classified as early event (T0 *n* = 30, T3 *n* = 16), whereas infants with the last maternal immunization event occurring between 12 months prior to birth and delivery were classified as late event (T0 *n* = 6, T3 *n* = 3) (Figure 3B). No statistically significant difference was shown either between or within the two cohorts at T0 and T3. Consequently, the timing of the last immunization event does not appear to influence the infant's immune protection.

### 3.4 Cytokine profiles in newborns enrolled

**FIGURE 4:**
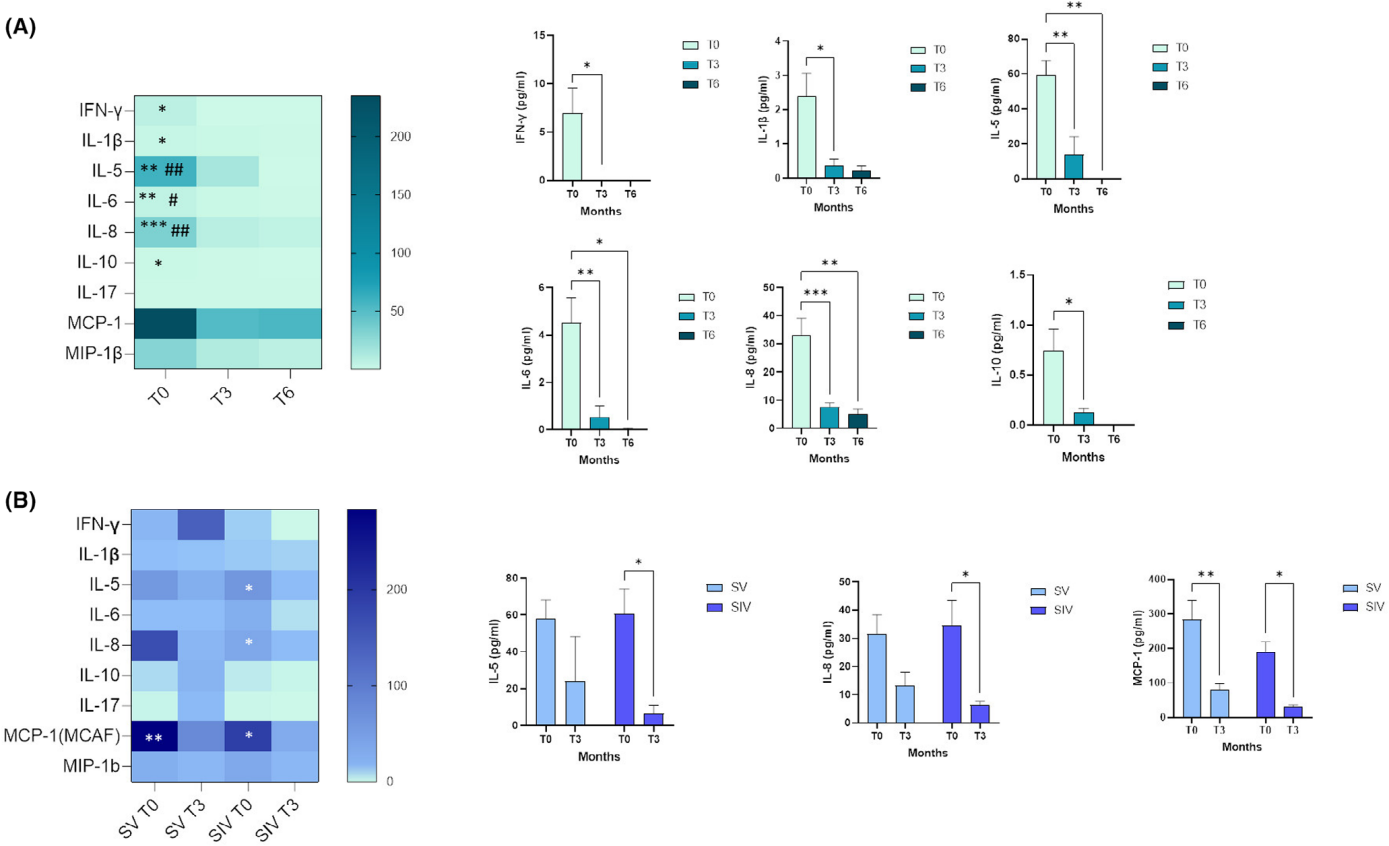
Cytokine and chemokine profile in newborns at birth, at 3 and 6 months of age. (A) Heatmap of cytokine/chemokine concentration (pg/mL) over time (T0, T3, and T6) in the plasma derived from all the newborns enrolled; mean values ± SEM are reported; significance was set at *p* < .05 (Two‐way ANOVA test, *p* values adjusted for multiple comparisons), * vs. T3: *p < .05, **p < .01, ***p < .001; # vs. T6: #*p* < .05, ##*p* < .01. Histograms of the significantly modulated cytokines/chemokines are reported. (B) Heatmap of cytokine/chemokine profile at different time points (T0 and T3) is shown by dividing the newborns born from SV (light blue) and SIV (dark blue) mothers; mean values ± SEM are reported; significance was set at *p* < .05 (Two‐way ANOVA test, *p* values adjusted for multiple comparisons), * vs. T0: *p < .05, **p < .01, ***p < .001. Histograms of the significantly modulated cytokines/chemokines are reported.

Plasma concentrations of cytokines and chemokines were measured in all available plasma samples at T0 (*n* = 38), T3 (*n* = 19), and T6 (*n* = 7). A strong pro‐inflammatory profile is present at birth but, as shown in Figure 4A, most of the molecules analyzed follow a significant reduction from T0 to T3, particularly IFN‐γ (T0 vs. T3: *p* < .05), IL‐1 β (T0 vs. T3: *p* < .05), IL‐5 (T0 vs. T3: *p* < .01; T0 vs. T6: *p* < .01), IL‐6 (T0 vs. T3: *p* < .01; T0 vs. T6: *p* < .05), IL‐8 (T0 vs. T3: *p* < .01; T0 vs. T6: *p* < .001), and IL‐10 (T0 vs. T3: *p* < .05).

As per the NA, the concentration of cytokines and chemokines in neonatal plasma was compared between the population of those born to mothers with a prior history of infection (SIV group, T0 *n* = 12, T3 *n* = 11) and those born to mothers only vaccinated against SARS‐CoV‐2 (SV group, T0 *n* = 11, T3 *n* = 8). Between T0 and T3, there was a significant decrease in MCP‐1 values in both groups (SV T0 vs. T3: *p* < .01; SIV T0 vs. T3: *p* < .05) and in IL‐5 (T0 vs. T3: *p* < .05) and IL‐8 (T0 vs. T3: *p* < .05) only in the SIV group (Figure 4B). No statistically significant differences emerged either by dividing the population by the number of immunizing events, as observed for neutralizing activity (data not shown).

## 4 DISCUSSION

Vaccinations for various infectious diseases (against Influenza, Tetanus, Diphtheria and acellular Pertussis – TDaP) have been well studied in terms of safety and efficacy for the protection of the newborn and are recommended in women before, during, and after pregnancy to reduce the burden of infections. However, widely variable estimates of effectiveness have been associated with vaccination in women,^15^, ^16^ which may be ascribed to the physiologic changes in pregnancy, including an immunosuppressed state. COVID‐19 vaccination has been shown to provide high protection against documented SARS‐CoV‐2 infection during pregnancy, comparable to that seen among the general population.^17^ Infants younger than 6 months of age are at high risk for complications of COVID‐19, including severe respiratory failure and death, and account for a disproportionately high percentage of hospitalizations among younger children. The effects of heterogeneous infection, vaccination, and boosting histories prior to and during pregnancy have not been extensively studied and are likely important for the protection of neonates.

Several studies support a causal link between maternal vaccination and protection against COVID‐19–related hospitalization among infants. A substantial body of evidence indicates that maternal antibodies against the SARS‐CoV‐2 virus, whether derived from vaccination or from a previous infection, are efficiently transferred to the infant during pregnancy or while breastfeeding. A recent report showed that infants whose mothers had been vaccinated during pregnancy had higher levels of anti–SARS‐CoV‐2 antibodies at 6 months of age than infants whose mothers had SARS‐CoV‐2 infection during pregnancy.^9^ However, in this study, mothers who experienced hybrid immunity were not included. It has been reported that SARS‐CoV‐2 vaccination and boosting, especially in the setting of previous infection, leads to significant increases in antibody levels and neutralizing activity even against the Omicron variants in neonatal cord blood,^18^ and maternal hybrid immunity was associated with a reduced risk of infant hospitalization for COVID‐19.^19^ In line with these results, we show that maternal neutralizing activity can be functionally passed to the offspring, and by subdividing our cohort based on the presence or absence of a previous maternal SARS‐CoV‐2 infection, neutralizing activity seemed to be higher in the context of a hybrid immunity. However, our key findings show that the number of immunizing events, more than the type of immunization (vaccination and natural infection alone or hybrid immunity), is crucial to the transfer of high neutralizing activity to neonates at birth, and that this maternal neutralizing activity in the plasma of infants is short‐lasting, as it has been detected up to 3 months from delivery. Despite the fact that the short‐lasting neutralizing activity does not appear to be associated with an elevated rate of hospitalization in the present cohort, the limited sample size and the absence of direct data on SARS‐CoV‐2 infection incidence preclude the drawing of definitive conclusions regarding the impact of our findings on clinical outcomes. For instance, studies on larger cohorts recommend booster vaccination of mothers no more than 14 weeks before the expected date of delivery.^2^ Another crucial modality of maternal antibody transfer to the offspring is breastfeeding. High levels of SARS‐CoV‐2‐specific IgA and IgG were found in stool from milk‐fed infants post‐maternal vaccination^20^ and SARS‐CoV‐2 neutralizing antibodies were detected in breastmilk from naturally infected women or those vaccinated with mRNA‐based vaccines,^21^ providing a foundation for passive immunization of breastmilk‐fed infants. Currently, we have limited evidence concerning the role of feeding mode within the first months of life in neonates. Our preliminary data on plasma from breastfed newborns, not shown here, support the evidence that breastfeeding is crucial to improve the persistence of SARS‐CoV‐2‐specific neutralizing activity up to 3 months from birth, an assumption reinforced by recent findings on vaccinated mothers.^2^, ^22^

Finally, our findings suggest that a pro‐inflammatory profile is detectable in the offspring from both SV and SIV mothers, independently from a previous maternal SARS‐CoV‐2 infection. Childbirth is an acutely stressful event that can cause many endocrine and immunological changes in both the woman and her offspring. Both inflammation and oxidative stress responses are enhanced during this period, acting as a crucial part in immune functions and neural development.^23^ For example, it has been reported that cytokines, such as IL‐1β and TNF‐α induce production of prostaglandins and metalloproteinases, promoting uterine contractions,^24^ cervical ripening^25^ and rupture of membranes.^26^ In summary, the profiles emerged from our results can be eventually considered a specific pattern of childbirth, rapidly normalized within 3 months from delivery.

## 5 CONCLUSIONS

Our study has some limitations, mostly related to the dropout of patients due to the decreased risk perception of the infection. Nevertheless, our findings show that the number of immunizing events, even more than hybrid immunity, can influence the magnitude of neutralizing activity in neonates at birth, despite a rapid decline within the first 6 months of age. As per this last consideration, a booster dose during pregnancy, especially during the late second or early third trimester, is recommended, as already suggested in previous studies.^2^, ^27^, ^28^, ^29^, ^30^, ^31^ However, despite the existing recommendation for maternal immunization against SARS‐CoV‐2, this recommendation is frequently not perceived as necessary by the mother. This is due to a lack of awareness of the necessity for infant protection during the first months of life. The underestimation of SARS‐CoV‐2 infection in infants can have adverse consequences for the child's health. It would therefore be beneficial for the recommendation of a booster dose during pregnancy to be upgraded to a “strongly recommended” status, given the urgency in conferring a higher and long‐lasting protection to the newborn.

## AUTHOR CONTRIBUTIONS

**Marta Stracuzzi:** Conceptualization; writing – original draft; writing – review and editing; validation; data curation. **Claudia Vanetti:** Conceptualization; writing – original draft; writing – review and editing; methodology; data curation; validation; formal analysis. **Micaela Garziano:** Methodology; writing – review and editing. **Maida Micheloni:** Writing – original draft; writing – review and editing; data curation; conceptualization. **Maria Luisa Murno:** Methodology; writing – review and editing. **Gian Vincenzo Zuccotti:** Writing – review and editing; funding acquisition; resources; validation. **Mario Clerici:** Funding acquisition; writing – review and editing; supervision; resources; validation. **Vania Giacomet:** Conceptualization; writing – original draft; writing – review and editing; visualization; validation; project administration; supervision; resources. **Daria Trabattoni:** Conceptualization; writing – original draft; validation; visualization; writing – review and editing; project administration; supervision; resources.

## FUNDING INFORMATION

The author(s) declare financial support was received for the research, authorship, and/or publication of this article. This study was partially supported by grants from Piano di Sostegno alla Ricerca UNIMI, Linea 2 and Fondazione Romeo and Enrica Invernizzi.

## CONFLICT OF INTEREST STATEMENT

The authors have no conflicts of interest to disclose.

### PEER REVIEW

The peer review history for this article is available at https://www.webofscience.com/api/gateway/wos/peer‐review/10.1111/pai.70084.

## ETHICS STATEMENTS AND INFORMED CONSENT

The study received approval from the ethical committee of the coordinating center in Milan (protocol number 0034645). All research was performed in accordance with relevant regulations, and informed consent was obtained from all the participants' parents or legal guardians. The study was performed in accordance with the Declaration of Helsinki.

## Supporting information


Figure S1.
