# Supplementary material for: Short‐lived neutralizing activity against SARS‐CoV‐2 in newborns of immunized mothers
Source: Pediatr Allergy Immunol. 2025 Apr 9;36(4):e70084. doi: 10.1111/pai.70084 (PMC11980968; doi:10.1111/pai.70084)
Supplement: Supplementary file 1 — Figure S1. [file PAI-36-e70084-s001.zip › Figure S1.docx]

Figure S1. Graph depicting all samples analyzed at T0, T3 and T6, with their respective neutralizing activity and maternal immunizing events in relation to the distance from the date of delivery.
