# Supplementary figures and images for: Short‐lived neutralizing activity against SARS‐CoV‐2 in newborns of immunized mothers
Source: Pediatr Allergy Immunol. 2025 Apr 9;36(4):e70084. doi: 10.1111/pai.70084 (PMC11980968; doi:10.1111/pai.70084)

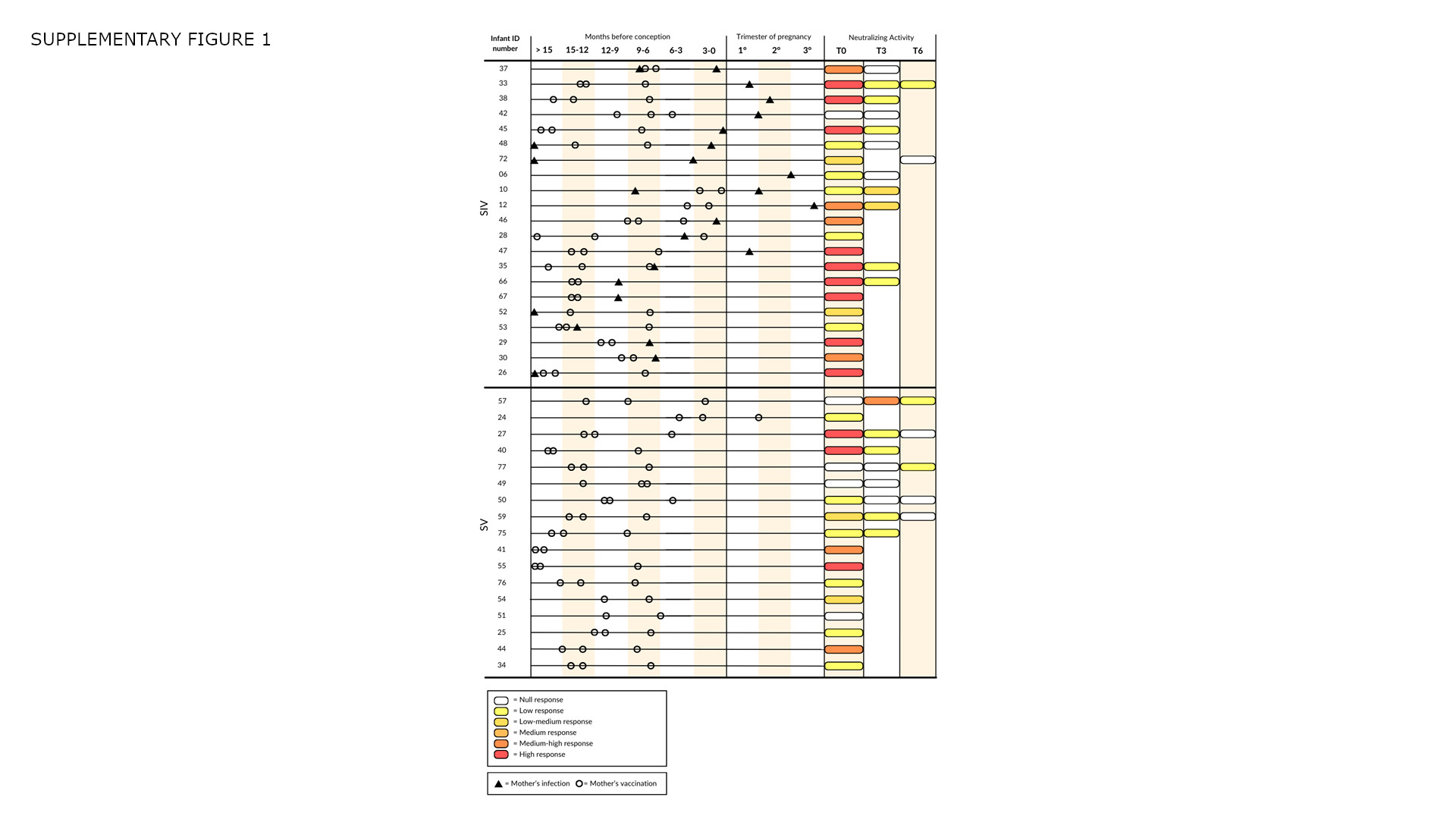

Supplement: Supplementary file 1 — Figure S1. [file PAI-36-e70084-s001.zip › pai70084-sup-0001-supinfo_Supplementary Figure 1.jpg]
